# Structural variation in the glycogen synthase kinase 3β and brain‐derived neurotrophic factor genes in Japanese patients with bipolar disorders

**DOI:** 10.1002/npr2.12083

**Published:** 2019-11-26

**Authors:** Yosuke Suga, Keiichiro Yoshimoto, Shusuke Numata, Shinji Shimodera, Shogo Takamura, Naoto Kamimura, Ken Sawada, Hiromitsu Kazui, Tetsuro Ohmori, Shigeru Morinobu

**Affiliations:** ^1^ Department of Neuropsychiatry Kochi Medical School Kochi University Nankoku Japan; ^2^ Watarigawa Hostipal Shimanto Japan; ^3^ Department of Psychiatry Course of Integrated Brain Sciences Medical Informatics Institute of Health Biosciences The University of Tokushima Graduate School Tokushima Japan; ^4^ Ginza Shimodera Clinic Tokyo Japan; ^5^ KOKORONO Support Center Kochi Health Sciences Center Ike Japan; ^6^ Department of Occupational Therapy School of Health Science and Social Welfare KIBI International University Takahashi Japan

**Keywords:** bipolar disorder, brain‐derived neurotrophic factor, copy number variation, glycogen synthase kinase 3^®^, lithium

## Abstract

**Background:**

Lithium is the first‐line drug for the treatment of bipolar disorders (BDs); however, not all patients responded. Glycogen synthase kinase (GSK) 3β and brain‐derived neurotrophic factor (BDNF) play a role in the therapeutic action of lithium. Since structural variations were reported in these genes, it is possible that these genomic variations may be involved in the therapeutic responses to lithium.

**Method:**

Fifty patients with BDs and 50 healthy subjects (mean age 55.0 ± 15.0 years; M/F 19/31) participated. We examined structural variation of the GSK3β and BDNF genes by real‐time PCR. We examined the influence of structural variation of these genes on the therapeutic responses to lithium and the occurrence of antidepressant‐emergent affective switch (AEAS). The efficacy of lithium was assessed using the Alda scale, and AEAS was evaluated using Young Mania Rating Scale.

**Results:**

Although we examined structural variations within intron II and VII of the GSK3^®^ gene and from the end of exon IV to intron IV and within exon IX of the BDNF gene, no structural variation was found in BDs. Whereas 5 of 50 patients exhibited three copies of the genomic region within exon IV of the BDNF gene, all healthy subjects had two copies. No difference in the therapeutic efficacy of lithium was found between patients with three and two copies. No difference in the occurrence of AEAS was found between the two groups.

**Conclusion:**

The amplification of the BDNF gene influenced neither the therapeutic responses to lithium nor the occurrence of AEAS.

## Abstract 2

Five of 50 patients with bipolar disorders exhibited three copies of the genomic region within exon IV of the BDNF gene. But, 50 healthy subjects had two copies. This amplification did not affect the therapeutic responses to lithium.

## Graphical abstract

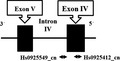

## 1 INTRODUCTION

Although it is well known that mood stabilizers, such as lithium and valproic acid, are effective in the treatment of bipolar disorders (BDs),^1^, ^2^, ^3^, ^4^, ^5^ a growing body of evidence shows that certain patients with BDs respond poorly to these 2 mood stabilizers.^6^, ^7^ Based on pharmacological studies of lithium and valproic acid, it has been suggested that a common therapeutic action of these mood stabilizers is the inhibition of glycogen synthase kinase (GSK) 3^®^ activity through increased serine phosphorylation.^9^, ^10^ In addition, chronic treatment with lithium and valproic acid has been reported to increase the levels of brain‐derived neurotrophic factor (BDNF) in rodent brain and human blood.^11^, ^12^, ^13^, ^14^, ^15^ Together, the inhibitory action of GSK 3^®^ and upregulation of BDNF are closely involved in the therapeutic mechanism of these mood stabilizers. In this context, it is hypothesized that structural genomic variations in the GSK 3^®^ or BDNF genes may affect the therapeutic efficacy of lithium and valproic acid. Patients with BDs who have structural genomic variations in these genes exhibit poor responses to lithium and valproic acid.

One type of structural genomic variation is copy number variation (CNV). The size of CNVs is greater than 1 kb, and different forms of CNVs such as deletions, duplications, insertions, and inversions have been reported. Based on the nature of CNVs, it is conceivable that CNVs can affect gene expression and gene function. Although the precise pathogenesis of bipolar disorder is unknown, several studies examining the contribution of CNVs to the pathogenesis of bipolar disorder have demonstrated BD‐associated CNVs.^16^, ^17^, ^18^, ^19^, ^20^, ^21^ For example, Green et al^18^ showed the possible contribution of CNV (duplication at 16p11.2) to BD using different array techniques. In addition, Ronai and associates^21^ reported a significant association between GSK 3^®^ CNV and BD using real‐time PCR. In contrast, to our knowledge, no study has demonstrated the contribution of BDNF CNVs to BDs, though large and rare CNVs are registered in the Database of Genomic Variants (DGV).

In this study, we first examined whether CNVs in the GSK 3^®^ and BDNF genes based on the DGV could be found in patients with BDs as assessed by quantitative real‐time PCR (qRT‐PCR) with TaqMan^Ⓡ^ Copy Number Assays (Applied Biosystems), and if found, we compared the prevalence of the CNVs between patients with BDs and healthy subjects. Second, we examined the influence of the CNVs found in this study on the age of onset in patients with BDs and subtypes of BDs. Third, we also examined the influence of CNVs on the therapeutic responses to lithium and antidepressant‐emergent affective switch.

## 2 MATERIALS AND METHODS

### 2.1 Subjects

Fifty patients with BDs (age: mean ± SD = 55.0 ± 15.0 years, gender: M/F = 19/31) and 50 age‐ and sex‐matched healthy subjects participated in this study. All subjects were Japanese. The characteristics of the patients are shown in Table 1. All patients were diagnosed by trained psychiatrists according to DSM‐IV‐TR criteria (American Psychiatric Association, 1994), on the basis of unstructured interviews and information from medical records. Forty‐seven patients with BDs received lithium treatment. The therapeutic response to lithium was evaluated using the Alda scale.^22^ Antidepressant‐emergent affective switch (antidepressant‐induced manic state) was evaluated based on the clinical course after administration of antidepressants. If patients showed manic symptoms (>12 points on the Young Mania Rating Scale^23^) within 1 month after the beginning of the addition of antidepressants to the lithium regimen, we assessed antidepressant‐emergent affective switch. Healthy subjects**,** free of any current or past psychiatric or physical diagnoses and any first‐degree relatives with bipolar disorders, were recruited by advertisement. This study was approved by the ethics committees of Kochi Medical School and University of Tokushima Graduate School. All subjects received a description of the study and gave written informed consent.

**Table 1:** Characteristics of the studied samples

	BDs (N = 50)	HS (N = 50)
Mean age (SD)	55.0 ± 15.0 y	55.0 ± 15.0 y
Age (min ~ max)	24 ~ 80 y	24 ~ 80 y
Male/female	19/31	19/31
Onset of BD	37.6 ± 14.5y	
BD subtype (N)	BD I: 31	
	BD II: 18	

Abbreviations: BD, bipolar disorder; HS, healthy subjects.

### 2.2 Selection of the genomic region in the GSK3β and BDNF gene

**Figure 1:**
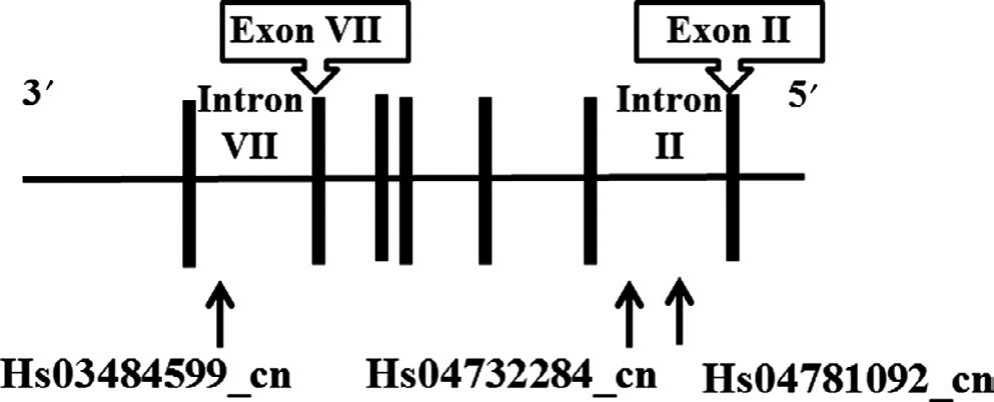
GSK3^®^ gene structure and the position of the PCR primers in this study. 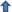: position of the PCR primers

With regard to CNVs in the GSK3^®^ gene, we focused on nsv829696 (Chr3: 119848821‐119992558, GRCh38:hg38) in the DGV. The region of this CNV (from intron II to intron IX) is relatively identical to a structural variant (variation_HU1; from 5ʹ‐noncoding region to exon IX) which was reported to be amplified in a patient with BD.^21^ Also, Ronai et al^21^ demonstrated amplification of the shorter region of the GSK3^®^ gene (from exon V to exon IV) in 7 patients with BD. We therefore examined structural variation within intron II and intron VII using real‐time PCR with TaqMan^Ⓡ^ Copy Number Assay (Hs04781092_cn, Hs04732284_cn, and Hs03484599_cn) (Figure 1).

**Figure 2:**
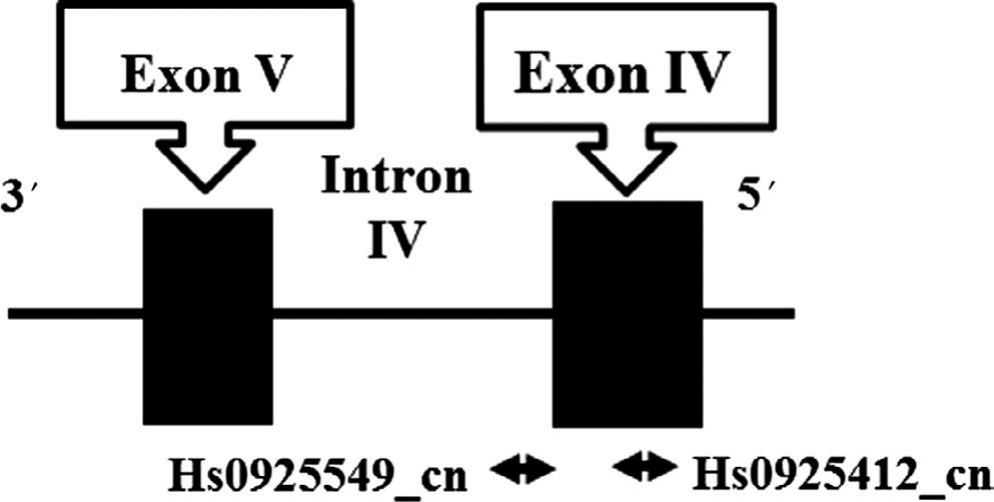
BDNF gene structure and the position of the PCR primers in this study. 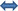: position of the PCR primers

In contrast with the GSK3^®^ gene, no study has demonstrated a contribution of BDNF CNVs to BDs. With regard to gene transcription in response to neuronal activation, it is well known that exon IV plays an important role in the transcription of this gene.^24^, ^25^, ^26^ In particular, Pruunsild et al demonstrated that BDNF exon I‐ and IV‐containing transcripts were closely involved in the transcription of the BDNF gene in the mouse cortical neurons expressing human BDNF gene.^25^ Especially, Yasuda et al^15^ demonstrated that lithium selectively increased the levels of exon IV‐containing BDNF mRNA in the cultured rat cortical neurons. In this context, since we focused on nsv95132 (chr11: 27720301‐27726000) covering the entire of exon VI that has been registered in the DGV, we used TaqMan^Ⓡ^ Copy Number Assay (Hs0925412_cn, and Hs0925549_cn) to examine CNVs in this region of nsv95132 (Figure 2). In addition, because it has been reported that exon IX is a transcribed exon, we also measured the CNVs in nsv832095 (chr11: 27538626‐27715739) covering the entire of exon IX (DGV) using TaqMan^Ⓡ^ Copy Number Assay (Hs01542529_cn).

### 2.3 Measurement of CNVs by a real‐time PCR

Blood samples (5 mL) were collected and placed in a vacuum tube containing heparin sodium and stored at −80°C. Genomic DNA (gDNA) was isolated using DNeasy^®^ Blood & Tissue Kits (Qiagen) according to the manufacturer's instructions.

The GSK 3^®^ and BDNF gene copy number were measured using a real‐time PCR system (7900HT Fast Real‐Time PCR System with a 384‐well plate; Applied Biosystems) using the TaqMan GSK 3^®^ and BDNF copy number assay (Applied Biosystems by Life Technologies) and TaqMan^Ⓡ^ copy number reference assay for human *RNase P* (Applied Biosystems, by Life Technologies). According to the manufacturer's instructions, 2 μL of gDNA (5 ng/μL) was added to the reaction mixture containing 5 μL of 2X TaqMan Genotyping Master Mix, 0.5 μL of 20 X TaqMan Copy Number Assay, 0.5 μL of 20X TaqMan copy number reference assay *RNase P*, and 2 μL of nuclease‐free water. The cycling conditions used were 10 min at 95°C, followed by 40 cycles of 15 s at 95°C and 60 s at 60°C. PCR was performed in duplicate.

StepOne Software v2.0 (Applied Biosystems by Life Technologies) was used for the post‐PCR plate read. Genotype and allele frequencies were subsequently determined by a simple counting method. The GSK3^®^ *and* BDNF copy number values for each sample were calculated using a relative quantitation algorithm with Applied Biosystems CopyCaller Software v2.0, (Applied Biosystems by Life Technologies), according to the manufacturer's instructions. The ΔCт of the test samples was compared with a calibrator of known copy number. A calibrator sample exhibiting 2 copies of the GSK3^®^ or BDNF gene was chosen. The quality metrics of the software (confidence and z‐score metrics) were used to validate the assigned copy number.

### 2.4 Statistical analyses

Significance in the occurrence of CNV between patients with BDs and healthy controls was estimated by chi‐square test. Gender difference, subtype (BP I and II) difference, and antidepressant response difference in the occurrence of the amplified type of the BDNF gene were also analyzed by chi‐square test. Effects of the amplified type of the BDNF gene on the onset of disorders and on the therapeutic responses to lithium in patients with BDs were examined by Mann‐Whitney *U* test. All statistical analyses were performed using SPSS Statistics 23 (IBM, Corp.).

## 3 RESULTS

According to the DGV, we measured CNVs within intron II and intron VII of the GSK3^®^ gene by real‐time PCR in patients with BDs. There were no CNVs in the GSK3^®^ gene in patients with BDs. Next, we measured the CNVs between exon IV and intron IV, and within exon IX of the BDNF gene in patients with BDs. Whereas 5 of 50 patients had three copies of the genomic region within exon IV, all patients had two copies of the genomic regions from the end of exon IV to intron IV of the BDNF gene. We then assessed CNV within exon IV by real‐time PCR in 50 healthy subjects, and we found no CNV in this region. There was a significant difference in the occurrence of the amplified type (CN = 3) within exon IV of the BDNF gene between patients with BDs and healthy subjects (Table 2). All patients exhibited two copies of the genomic region within exon IX.

**Table 2:** Occurrence of CNV within exon IV of the BDNF gene

Diagnosis	Wild type	Amplified type	*P* value
BDs	45	5	.029*
HS	50	0	

Note

Wild type: 2 copies, amplified type: 3 copies.

* *P* value by chi‐square test.

We next assessed the influence of the amplified type within exon IV of the BDNF gene on the clinical features of BDs. We found no significant difference in the occurrence of the amplified type between patients with BP I and BP II (Table 3). We also found no significant difference in the age of onset between patients with the amplified and wild‐type gene (Table 4).

**Table 3:** Occurrence of CNV within exon IV in the BDNF gene in patients with BDs

Diagnosis	Wild type	Amplified type
BD I	29	2
BD II	15	3

Note

No significant difference by chi‐square test.

**Table 4:** Influence of CNV within exon IV of the BDNF gene on the age of onset of BDs

CNV	n	Onset (y)
Wild type	45	37.6 ± 14.5
Amplified type	5	33.0 + 10.4

Note

No significant difference by Mann‐Whitney *U* test.

We then assessed whether the amplified type affected the therapeutic responses to lithium and antidepressants in this patient population. First, we evaluated the therapeutic response to lithium using the Alda scale and compared the responses between the amplified and wild type. There were no significant differences in the Alda scale A, B, or A‐B rates between these two groups (Table 5). Second, we compared the usage rates of combination therapy with lithium and antidepressants between these two groups and found no significant difference. In this study, all patients with the amplified type and 36 of 45 patients with the wild type were treated with combination therapy. For the 41 patients treated with combination therapy, we assessed the episode of the antidepressant‐emergent affective switch and compared the occurrence of the antidepressant‐emergent affective switch between patients with the amplified and wild type. There was no significant difference in the occurrence of antidepressant‐emergent affective switch between the two groups (Table 6).

**Table 5:** Effect of the CNV within exon IV of the BDNF gene on the therapeutic response to lithium in patients with BDs

Alda scale	Wild type	Amplified type
A scale	7.12 ± 2.35	7.40 ± 2.70
B scale	2.05 ± 1.68	2.60 ± 2.88
A‐B scale	4.88 + 3.59	4.80 + 5.50

Note

No significant difference by Mann‐Whitney *U* test.

**Table 6:** Influence of the CNV within exon IV of the BDNF gene on the treatment response to antidepressants

CNV	AEAS(+) (n)	AEAS(−) (n)
Wild	36	9
Amplified	4	1

Note

No significant difference by chi‐square test.

Abbreviation: AEAS, antidepressant‐emergent affective switch.

## 4 DISCUSSION

The results of the present study demonstrated that while no CNV was found in intron II or intron VII of the GSK3^®^ gene in 50 patients with BD, 5 of 50 patients with BD exhibited amplification (three copies) of the genomic region within exon IV of the BDNF gene. With regard to the copy number of the GSK3^®^ gene in BD, Ronai et al^21^ reported that 1 of 260 patients with BD had amplification of the genomic region covering the entire of the GSK3^®^ gene, and 5 of 260 patients had amplification of the genomic region from exon V of the GSK3^®^ to the NR1I2 gene 3ʹ region without any deletion of the GSK3^®^ 5ʹ region. The results of our study were not in agreement with the findings by Ronai et al.^21^ Although the reasons why we did not find any structural variation of the GSK3^®^ gene are unknown, differences in the sample sizes and patient ethnicities between the studies could be involved.

With respect to the relationship between structural variation of the BDNF gene and psychopathology, several studies have examined the association of BDNF haploinsufficiency with neurodevelopmental abnormalities in WAGR syndrome caused by 11p13 deletions near the location of the BDNF gene.^26^, ^27^, ^28^, ^29^, ^30^ A certain percentage of patients with WAGR syndrome exhibited deletion of the BDNF gene. For example, Han et al^27^ reported that WAGR patients with deletion of all or any portion of the BDNF gene had lower cognitive functioning and higher percentage meeting cutoff score for autism on the Autism Diagnostic Interview‐Revised scale as compared with WAGR patients did not have any deletion of the BDNF gene. Similarly, Egger and associates^31^ demonstrated the deletion of the genomic region within the BDNF opposite strand (chr 11p14.1) in autism spectrum disorder. With regard to the involvement of structural variation of the BDNF gene in the pathogenesis of mood disorders, Ernst et al^32^ demonstrated that two neurodevelopmental disorder patients with whole‐genome BDNF deletion have been diagnosed as having major depression.

In contrast with these previous studies, none of the participants in the present study were known to have a neurodevelopmental disorder. In this context, to our knowledge, this is the first study demonstrating the involvement of structural variation of the BDNF gene in the pathogenesis of BDs in the absence of a neurodevelopmental disorder. Five of 50 patients with BD exhibited amplification of the genomic region within exon IV of the BDNF gene. It is of interest that BDNF exon I‐ and IV‐containing transcripts were reported to be the most upregulated BDNF mRNAs in response to neuronal depolarization.^25^ Since it is well known that chronic administration of lithium increases the levels of BDNF in the brain,^33^, ^34^, ^35^ we hypothesized that this amplification may affect the therapeutic efficacy of lithium, and we compared the therapeutic responses to lithium between BD patients with and without the amplification, using the Alda scale.^22^ However, we failed to find any differences in the therapeutic efficacy of lithium between these two groups. In addition, it is also well known that regulation of BDNF expression is tightly involved in the therapeutic action of antidepressants. So, we next examined whether this amplification leads to the exaggerated response to antidepressants, in other words, antidepressant‐emergent affective switch. No significant difference in the occurrence of antidepressant‐emergent affective switch was found between these two groups. Based on these findings in our study, it appears that amplification of the small genomic region (27 701 498 ~ 27 701 399) within exon IV of the BDNF gene may be less important to regulation of BDNF transcription. In fact, Pruunsild et al^25^ showed that the induction of BDNF exon I‐containing transcript was much higher than that of BDNF exon IV‐containing transcript in response to neuronal depolarization.

There are several limitations of the present study that should be noted. First, the sample size in the present study was relatively small. In this context, we cannot rule out type II errors in the results demonstrating no significant statistical differences. In addition, all participants were from southern districts of Shikoku Island in Japan. Second, the analysis of the structural variation of the GSK3^®^ and BDNF gene in this study was conducted by using only a real‐time PCR method. More detailed analysis of structural variation using a comparative genomic hybridization (CGH) array or direct sequencing should be performed. Third, we did not measure the blood levels of BDNF exon IV mRNA or BDNF protein. These additional data could be helpful to determine whether amplification of the genomic region of exon IV of the BDNF gene affects BDNF gene. Thus, further studies using a much larger population and a different analytical method for structural variation in the BDNF gene should be undertaken to elucidate the involvement of BDNF gene CNV in the pathogenesis of BDs.

In summary, we found that 5 of 50 patients with BDs exhibited three copies of the genomic region within exon IV of the BDNF gene, while no amplification was seen in 50 healthy subjects. However, the amplification influenced neither the therapeutic responses to lithium nor the occurrence of antidepressant‐emergent affective switch. Further studies examining structural variation of the BDNF gene using real‐time PCR and CGH array may shed light on the pathogenesis of BD.

## CONFLICT OF INTEREST

The authors have no conflicts of interest to declare.

## AUTHOR CONTRIBUTIONS

SM designed this study and carried out real‐time PCR analysis, data analysis, and writing manuscript. YS reviewed the patients’ medical charts. YS, KY, SN, SS, ST, and NK diagnosed, treated, and evaluated patients. KS participated in the data analysis and preparation of the manuscript. HK and TO participated in the critical reading of the manuscript. All the authors have read and approved the final manuscript.

## DATA REPOSITORY

The raw data belonged to the present study cannot be made publicly available, because the disclosure of personal data was not included in the informed consent of the present study.

## APPROVAL OF THE RESEARCH PROTOCOL BY AN INSTITUTIONAL REVIEWER BOARD

This study was approved by the ethics committees of Kochi Medical School and University of Tokushima Graduate School.

## INFORMED CONSENT

All subjects received a description of the study and gave written informed consent.

## REGISTRY AND THE REGISTRATION NO. OF THE STUDY/TRIAL

n/a.

## ANIMAL STUDIES

n/a.
